# Supplementary material for: Acute pre-exercise hydrogen rich water intake does not improve running performance at maximal aerobic speed in trained track and field runners: A randomized, double-blind, placebo-controlled crossover study
Source: PLoS One. 2022 Dec 20;17(12):e0279307. doi: 10.1371/journal.pone.0279307 (PMC9767360; doi:10.1371/journal.pone.0279307)
Supplement: Attachment — Submitted filename: response_to_reviewers.docx [file pone.0279307.s004.docx]

**Reviewer #1**

The authors have addressed most of the comments raised in the previous review.

●	However, there is now a serious concern regarding the new title of the article that read, “Acute pre-exercise hydrogen-rich water intake had a possibly harmful effect on running performance at maximal aerobic speed in track and field runners.” The conclusion of “… a dose of 1,260 mL HRW ingested prior to an exhaustive run has a possibly harmful effect on the running performance at maximal aerobic speed in a cohort of the national level track and field runner. …” was reached by using the magnitude-based inference, albeit many other physiological variables had “most unlikely harmful” to “possibly beneficial” effects (Table 3). It seems that most of the physiological data do not justify the conclusion on the performance changes in time to exhaustion and distance to exhaustion in the current study. Further, there is no explanation on why hydrogen-rich water intake could be harmful to running performance when previous studies either reported no changes (e.g., references 11, 12, and 38) or an improvement (e.g., reference 39) in exercise time to exhaustion.

**Response:**

We reverted from magnitude-based inference to null hypothesis significance testing. Statistical analysis of the primary outcome, time to exhaustion, resulted in a nonsignificant difference between HRW and placebo (*p* = 0.20) with a trivial effect size (Cohen’s d = -0.19). The current title is: “Acute pre-exercise hydrogen rich water intake does not improve running performance at maximal aerobic speed in trained track and field runners”.

●	Line 380-386: It is interesting to learn that the optimal sample size for magnitude-based inference should be 50 participants (based on Hopkins and Batterham, 2016). However, the authors obtained a decisive result that HRW had a possibly harmful effect on time to exhaustion with only half of the optimal sample size … how could this result be possibly decisive or valid? Furthermore, did the previous studies (references 2, 4, and 8) that used even smaller sample sizes (of 10, 8, and 12, respectively) also use the magnitude-based inference method?

**Response:**

Since the null hypothesis significance testing was returned, the original power analysis associated with it was also returned (line 211). The minimal sample size resulted in 24 participants. Null hypothesis significance testing was used in all references 2, 4 and 8.

**Reviewer #2**

Thanks again for another conscientious rewrite. I'm really sorry to come back to you with more concerns. I've paid more attention to the physiology this time, and I think you should present MBI in a way that is more reader-friendly.

●	In your response to me, and at Line 33, you wrote "the effect was possibly (67.7%) harmful…" Percentages >9.9 do not normally need a decimal place. To provide one here implies an unrealistic precision of estimation of the percent. Make it 68%. Or just say that the effect was small, possibly harmful and very unlikely beneficial, without the percentages.

**Response:**

The chances calculated using magnitude-based inference was removed in the current manuscript. However, means, standard deviations, and p-values were rounded as recommended in Hopkins WG, Marshall SW, Batterham AM, Hanin J. Progressive statistics for studies in sports medicine and exercise science. Med Sci Sports Exerc. 2009;41(1):3–13.

●	You responded "The only exception is normality testing, for which we found no alternative for magnitude based inference." The reason there is no alternative in MBI is because normality testing is just plain wrong, as explained in the 2009 MSSE article on progressive statistics. In the manuscript at Lines 186-187 you have "The normality of log-transformed data was verified using the Shapiro-Wilk test and p < 0.05 was considered statistically significant." Well, if you have a small sample size, you could have obvious non-normality that gives p>0.05 for this test; if you have a large sample size, you could have data that look beautifully normal, but you can get p<0.05. In fact, with a large-enough size, you will ALWAYS get p<0.05 for the nil hypothesis for any statistic you like to name. So that shows that "testing" does not "verify" normality, and is yet another example of the failure of NHST. But in any case, normality of the original data (or more properly, the residuals in the linear model, or the residuals and random effects in a mixed linear model) is simply not an issue, thanks to the central limit theorem. The real issue is UNIFORMITY of errors and effects, which is why we use log or other transformations, when necessary: we do it not to achieve normality but to achieve uniformity. So please remove Lines 186-187 and 224-228.

**Response:**

Since the null hypothesis significance testing was returned, the results of the Shapiro-Wilk test were retained. In addition, we visually inspected the quantile-quantile plot and no serious departures from normality were found. The logarithmic transform used to achieve uniformity was retained in the current manuscript.

●	Lines 33-35 and wherever in the body of the manuscript… I don't ever talk about a beneficial or harmful correlation, and I have said in various places that covariates should be assessed non-clinically. The only time you would use clinical MBI for a covariate is when you estimate the effect of the treatment implemented with subjects who have a high value of the covariate, and ditto with a low value of the covariate, but that's not evaluating the covariate as such, and I don't expect you to do that at this stage. Instead, just state at Line 33 that "There was little observed correlation between the effect of HRW and placebo time to exhaustion (r=-0.04, 90% confidence interval -0.38 to 0.31)." I would expect a more negative correlation due to regression to the mean with TTE, so if anything there might be a tendency towards a less harmful or even a beneficial effect of HRW with longer times to exhaustion, once regression to the mean is factored out, but don't worry about it. You need error of measurement to estimate regression to the mean.

**Response:**

Also, for the correlation analysis, we reverted to null hypothesis significance testing. We know the regression to the mean, but we did not quantify it. We agree with you that we would need an estimate of measurement error, which is not possible to obtain from a simple crossover study.

●	I see you got higher correlations for some subject characteristics. Why didn't you say something about them in the Abstract in relation assessing any individual responses? Something like this, without mention of specific characteristics and correlations: "Correlations between the effect of HRW and subject characteristics that could explain any individual responses were indecisive." I see you haven't discussed that issue in the manuscript. Why not? What's the point of showing all those correlations if you don't say anything about them?

**Response:**

The result of the correlation analysis was added to the abstract (line 39). The role of potential moderators was discussed in the discussion section beginning on line 332.

●	Line 181 ff. You don't say whether you adjusted for an order effect. The runners were not familiarized with the TTE test, so presumably on average they ran longer in the second test. Adjustment for an order effect might improve the precision of the estimates.

**Response:**

We agree with you that actual familiarization, i.e., one run at maximal aerobic speed before the experimental part, was not performed in this study. Performing such familiarization is not easy due to the tight training and racing schedule. However, participants completed an incremental protocol to obtain maximal aerobic speed. In addition, participants have experience with the treadmill VO2max test as part of their duties. Therefore, we did not consider participants to be naive.

●	Lines 210-211. "Smallest worthwhile change for correlation coefficient was set at 0.3…" It should read "The smallest important correlation coefficient was…" But see my next comments.

**Response:**

This comment is no longer relevant because magnitude-based inference was abandoned.

●	Lines 210-217. I got lost here with what you were considering to be the two variables in the correlations. Table 4 shows only time to exhaustion as the dependent and all the subject characteristics (including physiological measures in the placebo condition) as moderators, and changes in physiological measures as mediators. Make that clear in this section of the Methods.

**Response:**

The sentence from the previous manuscript may be hard to read because it describes the correlation calculation published in paper (7). This sentence was removed from the current manuscript.

●	Lines 212-213. "We consider a correlation coefficient with a magnitude <0.3 to be impractical for prediction purposes." Really? Why? The only logical way to assess the magnitude of the linear relationship between a subject characteristic (a potential moderator) and an athletic performance measure is to consider the magnitude of the effect of two between-subject standard deviations of the characteristic; that is, you multiply the slope (of the performance change vs the characteristic) by 2 SD of the characteristic, then evaluate how big that is, using the scale you have already mentioned for time to exhaustion (smallest important is 0.3 of 10%). What you are thereby estimating is the effect on a subject who is 1 SD above the mean minus the effect on a subject who is 1 SD below the mean. The same approach applies to estimating the magnitude of the effect of a mediator: the effect of 2 SDs of the change score. Really, though, for a potential mediator, what's more important is two estimates: the effect due to the mediator (the slope in the delta-delta graph times the mean change in the mediator) and the effect independent of the mediator (the intercept in the delta-delta graph, i.e., where the change in the mediator is zero). But don't go there at this late stage, especially considering you did not get substantial change in mean performance for a mediator to explain! It all seems too difficult, doesn't it? That doesn't mean you can just make up a smallest important correlation without justification. In fact there is a justification. You can derive a value for the smallest important correlation for use throughout Table 4 by working backwards from the smallest important in the mean. (In what follows, I had a Greek Delta symbol that is rendering as a box.) The equation of a regression line is ΔY/SDY = r. ΔX/SDX, where ΔY is the mean of Y minus Y, and ditto X. You want the effect of 2SDX. Therefore, setting ΔX to 2SDX, we get ΔY/SDY = r.2SDX/SDX = 2r. If you set ΔY to the smallest important (you justified 3% for TTE), and given (from Table 3) SDY = 100*53/227 = 23%, then the smallest important correlation r = 3/23/2 = 0.06. These calculations should be done with log-transformed factor effects and SDs, giving the smallest important r = 0.3*log(1.10)/log(1.23)/2 = 0.07. Thresholds for moderate, large, very large and extremely large are given by replacing 0.3 by 0.9, 1.6, 2.5 and 4: correlations of 0.20, 0.36, 0.57 and 0.91. (By the way, this equation for the straight line can be used to show that a correlation of 0.1 corresponds to a Cohen's d of 0.2.)

**Response:**

Thank you for the detailed explanation of SWC for correlation. However, we decided to abandon magnitude-based inference, so the SWC value is no longer needed.

●	Lines 213-217. "A possibly negative correlation (r = −0.54, 90% confidence interval −0.79 to −0.15) was found between the effect of HRW on race time and pooled race time (7). Therefore, negative values of the correlation coefficient were considered beneficial in the analysis of performance related variables (time to exhaustion, maximal oxygen consumption, maximal power output, and maximal aerobic speed)." There are several wrong things here. First, you appear to be accepting this published negative correlation as definitive for a modifying effect of race time on the effect of HRW on race time. I haven't read Reference 7, but I presume that the negative correlation is confounded by regression to the mean. In fact, surely it's practically all regression to the mean, because otherwise this correlation is pretty good evidence that HRW does have a beneficial physiological effect for slower runners. Secondly, I can’t get my head around the logic of "therefore". See what I say earlier about beneficial and harmful correlations. Thirdly, the published correlation is for race time, which is similar to time to exhaustion (larger is worse) but opposite to those other measures of performance (larger is better), so it's all too confusing. Again, see above. In short, don't talk about beneficial and harmful correlations.

**Response:**

We agree that the use of clinical magnitude-based inference is not appropriate here. The solution is to use non-clinical magnitude-based inference. However, we decided to return to null hypothesis significance testing.

●	I see in the first paragraph of the Discussion you talk about an hypothesis of benefit when you undertook the study. Maybe it's worth pointing out at the end of that paragraph that your finding that the effect was very unlikely beneficial represents rejection of that hypothesis at the 5% level (for those readers who like to assess outcomes in terms of rejection of hypotheses).

**Response:**

The very unlikely beneficial result of magnitude-based inference is not equivalent to rejecting the hypothesis at the α = 0.05 level. The SWC would have to be set to zero for the abovementioned statistical method to be equivalent. Therefore, we did not follow this recommendation.

●	Lines 326-328. "Hopkins and Batterham (40) showed that the optimal sample size for magnitude based inference should be 50 participants. In this study, only half of the optimal sample size was used." The optimum (better: minimum desirable) sample size depends on the smallest important, error of measurement, and design, all of which are different in your study from the simulations of Hopkins and Batterham. There is no "one (sample) size fits all", except for a simple comparison of means in two groups, using standardization to assess magnitude of the difference in means.

**Response:**

This text was removed, and classical power analysis was returned.

●	Table 3. The time to exhaustion was a little over 3.5 min. Obviously it was a supra-VO2maximal exercise test, at least in terms of target intensity. Three minutes, especially following a warm-up, is enough time to reach a plateau in VO2max. So how come the mean VO2 in this table is only ~50 ml/min/kg and not the 55 achieved in the VO2max test? Or have you averaged the VO2 over the whole time-to-exhaustion test? If so, say so clearly, and explain why. If so, it's more of an oxygen-cost measure. You haven't described or discussed its Δ-Δ relationship with TTE in those terms. Also, the clearest and largest correlation was between Δlactate post-exercise and ΔTTE, but you haven't discussed it. It's what you'd expect, if the runners varied between tests in their near-maximal effort in the TTE test: when they made a bigger effort, they ran longer and their lactate was higher.

**Response:**

Thank you for this comment. Previously, ventilation parameters were calculated for the entire running at maximal aerobic speed. This was erroneously described as parameters calculated from the last 30 s. In the current manuscript, we report the correct values calculated from the last 30 s. This is a study evaluating the effect of HRW on performance and physiological variables. Therefore, correlations Δ versus Δ are not appropriate for such an article. They are more appropriate for a reliability study evaluating differences between tests or for a study describing the physiological determinants of running. We have added these correlations based on your request from the previous round of review. We see that they did not provide any new information regarding HRW, so we decided to remove them from the current manuscript.

●	Tables 3 and 4 are far too busy with the decision text you've copied from the spreadsheet. "Get more data." "Do not use." "Use." Look, readers want to know whether or not there are effects. More precisely, they don't want to be distracted by effects that "could be" (this has specific meanings) substantial positive and negative (or beneficial and harmful), and they want their eye to be drawn more to those effects where the evidence is stronger, either for substantial or for trivial. So over more than 20 years, Hopkins and his colleagues have gradually worked out how to do that. Did you look to see how they have done it, especially recently in the light of the changes arising from the attacks by Sainani et al.? Apparently not. Please have another go at this table, this time departing from the approach others have taken only if you can justify it.

**Response:**

We followed your guidelines published in Batterham AM, Hopkins WG. Making meaningful inferences about magnitudes. Int J Sports Physiol Perform. 2006;1(1):50–7 and implemented in your latest spreadsheet downloaded from the Sportscience website. We were unable to find any update to the guidelines or the spreadsheet. We are aware of many papers that use magnitude-based inference and have not found a more recent approach other than reporting effect sizes alongside results of magnitude-based inference. We find magnitude-based inference interesting because it can distinguish between a trivial effect and an unclear effect, which traditional null hypothesis significance testing cannot. However, magnitude-based inference also has several shortcomings that have not been resolved to date. One of them is the increase in type 1 statistical error (Lohse KR, Sainani KL, Taylor JA, Butson ML, Knight EJ, Vickers AJ. Systematic review of the use of "magnitude-based inference" in sports science and medicine. PLOS ONE. 2020;15(6):e0235318). Therefore, after a heated discussion in the authors' collective, we finally decided to return to null hypothesis significance testing. This manuscript is an example. In the current version, using null hypothesis significance testing, the result for time to exhaustion is *p* = 0.20, *d* = -0.19, which can be interpreted as a non-significant and trivial difference between HRW and placebo. On the other hand, magnitude-based inference interpreted this as a possibly harmful effect. We believe that this is just a consequence of the inflation of the type 1 statistical error in magnitude-based inference. We did not want to give up on assessing the effect using the smallest worthwhile change, so we provided an assessment of individual responses, but did so classically using a chi-square test (Figure 3).

●	Don't forget to say and do something about the increase in inferential errors that you've got from the large number of effects. You've made an attempt at Lines 378-380: "Multiple variables were compared, and no technique was used to control for Type 1 statistical error. The results should therefore be interpreted with caution." What's wrong here? First, you weren't comparing variables. Secondly, the main pre-planned effect in this and any study is not subject to inflation of error. Thirdly, there are ways of reducing errors with multiple effects, and you should have used one. And finally, exactly what does it mean when you state "results should be interpreted with caution"?

**Response:**

This limitation was added at the request of Reviewer 3 from the previous round of review. In the current manuscript, this limitation was rephrased to “Several variables were compared, and no technique was used to control for Type 1 statistical error.”

●	In Table 3, the only obvious clinically relevant effect is time to exhaustion. Distance to exhaustion would have exactly the same smallest important, so it's not even worth showing. The others would be done clinically, only if you can argue that a change in a particular direction (positive or negative) would be related to performance. For example, what about HRmax? If the treatment produced a higher HRmax, would you expect that be associated with better performance? Maybe, because being able to reach a higher max heart rate would imply more cardiac output, other things being equal, but you seem to have assumed the opposite. Lactate pre, I have no idea. Lactate post, yes, as explained above, and for the same reason as HRmax, but you've assumed the opposite. The submax (or whatever they are) physiological measures? An increase in those might be expected to be associated with impaired performance, which you have apparently assumed, but probably not for physiological reasons. Maybe you should assess all effects in Table 3 non-clinically, apart from TTE. Table 4 needs to be entirely non-clinical. And how about assessing the observed magnitudes of effects as trivial, small, moderate and so on? Don't readers want to know how strong the effects were?

**Response:**

In addition to returning to null hypothesis significance testing, we added an effect size calculated as Cohen's d. We also classified the effect size according to the thresholds published in Hopkins WG, Marshall SW, Batterham AM, Hanin J. Progressive statistics for studies in sports medicine and exercise science. Med Sci Sports Exerc. 2009;41(1):3-13.
