# Supplementary material for: Acute pre-exercise hydrogen rich water intake does not improve running performance at maximal aerobic speed in trained track and field runners: A randomized, double-blind, placebo-controlled crossover study
Source: PLoS One. 2022 Dec 20;17(12):e0279307. doi: 10.1371/journal.pone.0279307 (PMC9767360; doi:10.1371/journal.pone.0279307)
Supplement: Attachment — Submitted filename: Reviewer report (17-07-22).docx [file pone.0279307.s005.docx]

Title: Acute pre-exercise hydrogen rich water intake does not improve running performance at maximal aerobic speed in trained track and field runners

GENERAL COMMENTS

This study investigated the effects of acute, pre-exercise, hydrogen-rich water (HRW) ingestion on running time to exhaustion at maximal aerobic speed in trained track and field runners. I have read with attention the previous reviewers’ comments. I feel that the study is interesting, but however, I found that there are several challenges in carry out other studies, due to a prori no results may not directly to indicate that exercise hydrogen-rich water intake does not improve running performance, it was evaluated at a particular condition (maximal aerobic speed), but not at anaerobic speed, and with a “time” as a performance marker, but not at other markers (efficiency of running, glycogen content, fat, CHO etc), and with specific sample characteristics (young runners). Additionally, the study protocol and design it is high-quality, and easy to replicate, then I like to recommend the study for future other studies that can help us to carry out other more complex studies.

ABSTRACT

It is well design

INTRODUCTION

-I like to suggest to the authors to include a little more information regarding that pre-exercise H2 ingestion have been reported not at all exercise modalities, intensities, volumes, nor including other outocmes more thatn the “time” in a performance, and population (runners trained).

Methods

I found the methods reported in a very good quality

The study protocol figure is very understandable

Statistical analyses are very clear.

RESULTS

There are very clear described.

DISCUSSION

-I like to suggest to the authors to include some future potential other study conditions as I have stated previously (i.e., other populations), where this previous study give us only a high-quality, but very specific condition in which the hydrogen rich water (HRW) ingestion do not add new advantages.

Tables

Are very clear

Figures

Are very clear

References

Are very up-dated.
