# Supplementary material for: Acute pre-exercise hydrogen rich water intake does not improve running performance at maximal aerobic speed in trained track and field runners: A randomized, double-blind, placebo-controlled crossover study
Source: PLoS One. 2022 Dec 20;17(12):e0279307. doi: 10.1371/journal.pone.0279307 (PMC9767360; doi:10.1371/journal.pone.0279307)
Supplement: Attachment — Submitted filename: response_to_reviewers.docx [file pone.0279307.s006.docx]

**Journal Requirements**

●	Please ensure that your manuscript meets PLOS ONE's style requirements, including those for file naming.

**Response:** We carefully read the PLOS ONE Submision Guidelines and prepared the manuscript in accordance with these guidelines.

●	Please review your reference list to ensure that it is complete and correct. If you have cited papers that have been retracted, please include the rationale for doing so in the manuscript text, or remove these references and replace them with relevant current references. Any changes to the reference list should be mentioned in the rebuttal letter that accompanies your revised manuscript. If you need to cite a retracted article, indicate the article’s retracted status in the References list and also include a citation and full reference for the retraction notice.

**Response:** The list of references was carefully checked. No retracted papers were identified.

**Reviewer #1**

A randomized placebo-controlled crossover research study aimed to investigate the effects of ingesting hydrogen rich water (HRW) on running time in track and field runners (n=24). Time to exhaustion, post-exercise blood lactate concentration, maximal heart rate, and oxygen uptake were not significantly different between placebo and HRW.

1– Abstract: Define r.

**Response:** Thank you for your comment. The abbreviation was replaced with the full title and the sentence now reads “Spearman’s correlation coefficients ranged from −0.28 to 0.30, all p ≥ 0.16”.

2– Table 1: Indicate if the distribution of the variables were checked for normality. For nonparametric data, summarize using median, first and third quartiles.

**Response:** Thank you for this suggestion. It is our mistake that we neglected to check the normality assumption. We added the results of the Shapiro-Wilk test. Three variables were significantly different from the normal distribution. We therefore added median, first and third quartile values to Table 1. In the correlation analysis we switched to a non-parametric alternative – Spearman’s correlation coefficient.

3– Line 229: Within the text, indicate the statistical method used to estimate the correlations. Since the p-value tests the null hypothesis that the correlation equals zero, it may be more precise to state that the correlations were not statistically different than zero.

**Response:** We re-read the manuscript and found that there was no clear distinction between the analysis of individual responses and correlation analysis. We rearranged the text and hope this helped to make it clearer. Spearman’s correlation coefficient as the statistical method used was added to the text. Thank you for your comment regarding the null hypothesis of zero correlation. We rewrote the sentence. Please see the Results section in the manuscript.

**Reviewer #2**

GENERAL COMMENTS

This study investigated the effects of acute, pre-exercise, hydrogen-rich water (HRW) ingestion on running time to exhaustion at maximal aerobic speed in trained track and field runners. I have read with attention the previous reviewers’ comments. I feel that the study is interesting, but however, I found that there are several challenges in carry out other studies, due to a prori no results may not directly to indicate that exercise hydrogen-rich water intake does not improve running performance, it was evaluated at a particular condition (maximal aerobic speed), but not at anaerobic speed, and with a “time” as a performance marker, but not at other markers (efficiency of running, glycogen content, fat, CHO etc), and with specific sample characteristics (young runners). Additionally, the study protocol and design it is high-quality, and easy to replicate, then I like to recommend the study for future other studies that can help us to carry out other more complex studies.

ABSTRACT

It is well design.

INTRODUCTION

I like to suggest to the authors to include a little more information regarding that pre-exercise H2 ingestion have been reported not at all exercise modalities, intensities, volumes, nor including other outocmes more thatn the “time” in a performance, and population (runners trained).

METHODS

I found the methods reported in a very good quality.

The study protocol figure is very understandable.

Statistical analyses are very clear.

RESULTS

There are very clear described.

DISCUSSION

I like to suggest to the authors to include some future potential other study conditions as I have stated previously (i.e., other populations), where this previous study give us only a high-quality, but very specific condition in which the hydrogen rich water (HRW) ingestion do not add new advantages.

Tables

Are very clear.

Figures

Are very clear.

References

Are very up-dated.

**Response:** Thank you for your comments and suggestions, particularly relating to the Introduction and Discussion.

Regarding the Introduction, we followed your suggestions, and some of the sentences were rewritten in order to be more specific and explanatory. In addition, we also added the results of some very recent studies that shed more light on the specific exercise intensities, where H2 showed an ergogenic effects. Please, see lines 57–68.

Relating to our approach in the writing of the Discussion, our main goal, particularly in this study, was to investigate whether pre-exercise hydrogen rich water (HRW) intake using our specific hydration strategy (time, dose, chemical properties of HRW) improved running time to exhaustion in very specific population, i.e. trained field and track athletes. To date, we have published six H2 studies in relation to different modes of exercise, intensity level and population. For instance, one study investigated the effect of H2 inhalation on the functional state of post-COVID-19 patients. However, the take home message of the present study was to show coaches that if they decided to use HRW immediately before the race in well trained athletes, HRW does not work well in terms of the expected improvement in running performance across distances from 1500 m to 3000 m. We absolutely agree with your opinion to design future studies that investigate different physiological variables, biomarkers, and population etc. When working with well-trained athletes, there are many practical issues that need to be considered. For example, the research needs to fit around the athletes‘ training programmes and cannot interupt their schedule too much. Nevertheless, our study includes many references to previous studies showing the positive effects of H2 supplementation on fitness, performance, physiological and psychometric responses.
