# Supplementary material for: Acute pre-exercise hydrogen rich water intake does not improve running performance at maximal aerobic speed in trained track and field runners: A randomized, double-blind, placebo-controlled crossover study
Source: PLoS One. 2022 Dec 20;17(12):e0279307. doi: 10.1371/journal.pone.0279307 (PMC9767360; doi:10.1371/journal.pone.0279307)
Supplement: Attachment — Submitted filename: response_to_reviewers.docx [file pone.0279307.s007.docx]

●	Please review your reference list to ensure that it is complete and correct. If you have cited papers that have been retracted, please include the rationale for doing so in the manuscript text, or remove these references and replace them with relevant current references. Any changes to the reference list should be mentioned in the rebuttal letter that accompanies your revised manuscript. If you need to cite a retracted article, indicate the article’s retracted status in the References list and also include a citation and full reference for the retraction notice.

**Response:** We used the Mendeley reference manager to prepare the reference list. We synchronized all available records with the MEDLINE database. No retracted papers were identified. Therefore, we believe that our reference list is correct. If it is not, please indicate the occurrence of the incorrectness.

●	Reviewer #3: The manuscript written in well organized manner, design of the study well constructed, only one comment related to manuscript title as it should contain research type.

**Response:** Thank you for this suggestion. We added the following words to the title: “A randomized, double-blind, placebo-controlled crossover study”. We looked at the titles of articles in PLOS ONE to see how this type of study is most often writen. We found that a hyphen and a comma are very often used. We therefore rewrote the occurences of this phrase in the abstract and at the beginning of the method section.
